# When the Embryo Meets the Endometrium: Identifying the Features Required for Successful Embryo Implantation

**DOI:** 10.3390/ijms25052834

**Published:** 2024-02-29

**Authors:** Valentina Lacconi, Micol Massimiani, Ilenia Carriero, Claudia Bianco, Carlo Ticconi, Valentina Pavone, Alessandra Alteri, Ludovico Muzii, Rocco Rago, Valerio Pisaturo, Luisa Campagnolo

**Affiliations:** 1 Department of Biomedicine and Prevention, University of Rome Tor Vergata, Via Montpellier 1, 00133 Rome, Italy; valentina.lacconi@uniroma2.it (V.L.); ilenia.carriero@alumni.uniroma2.eu (I.C.); claudia.bianco@students.uniroma2.eu (C.B.); 2 Saint Camillus International University of Health Sciences, Via di Sant’Alessandro 8, 00131 Rome, Italy; micol.massimiani@unicamillus.org; 3 Department of Surgical Sciences, Section of Gynaecology and Obstetrics, University of Rome Tor Vergata, Via Montpellier 1, 00133 Rome, Italy; ticconi@uniroma2.it; 4 Reproductive Sciences Laboratory, IRCCS San Raffaele Scientific Institute, 20132 Milan, Italy; pavone.valentina@hsr.it; 5 Obstetrics and Gynaecology Unit, IRCCS San Raffaele Scientific Institute, 20132 Milan, Italy; ale.alteri@libero.it; 6 Department of Maternal and Child Health and Urological Sciences, “Sapienza” University of Rome, Policlinico Umberto I, 00161 Rome, Italy; Ludovico.muzii@uniroma1.it (L.M.); valerio@pisaturo.com (V.P.); 7 Physiopathology of Reproduction and Andrology Unit, Sandro Pertini Hospital, Via dei Monti Tiburtini 385/389, 00157 Rome, Italy; rocco.rago@aslroma2.it

**Keywords:** IVF, endometrial receptivity, implantation, blastocyst, embryo, ART, AI, extracellular vesicles, transcriptomic signature, secretome

## Abstract

Evaluation of the optimal number of embryos, their quality, and the precise timing for transfer are critical determinants in reproductive success, although still remaining one of the main challenges in assisted reproduction technologies (ART). Indeed, the success of in vitro fertilization (IVF) treatments relies on a multitude of events and factors involving both the endometrium and the embryo. Despite concerted efforts on both fronts, the overall success rates of IVF techniques continue to range between 25% and 30%. The role of the endometrium in implantation has been recently recognized, leading to the hypothesis that both the “soil” and the “seed” play a central role in a successful pregnancy. In this respect, identification of the molecular signature of endometrial receptivity together with the selection of the best embryo for transfer become crucial in ART. Currently, efforts have been made to develop accurate, predictive, and personalized tests to identify the window of implantation and the best quality embryo. However, the value of these tests is still debated, as conflicting results are reported in the literature. The purpose of this review is to summarize and critically report the available criteria to optimize the success of embryo transfer and to better understand current limitations and potential areas for improvement.

## 1. Introduction

Implantation in mammals relies on the activation of spatially and temporally regulated signals from both the embryo and the endometrium. Synchronization of the embryo-endometrium dialogue represents a limiting step for a successful pregnancy, and the rate of clinical pregnancy in normal cycles only reaches about 30–40% [1]. Despite the improvement of IVF techniques and preimplantation genetic testing (PGT) to assess embryo euploidy, implantation failure remains a challenge. Approximately 10–30% of patients referred to IVF clinics experience implantation failure [2], and attention has been focused on measures to improve pregnancy outcomes. Among these, the identification of difficult procedures of embryo transfer [3], methods of creating optimal embryo cultures [4,5], the standardization of morphological criteria to classify blastocyst competency [6,7], the improvement of PGT [8], and measures to identify the optimal day for embryo transfer [9,10,11], have been reported. Nevertheless, despite efforts in both endometrial analysis and embryonic evaluation, our capacity for assessment remains limited. One of the main complications in assisted reproductive techniques is recurrent implantation failure (RIF), defined as the failure to achieve pregnancy after the transfer of at least 3 good-quality embryos [12]. While embryo quality can be assessed using morphological and molecular parameters, the evaluation of proper endometrial competency/receptivity is more challenging. Due to its role in limiting embryo implantation, the concept of the endometrium as the guardian of pregnancy has been proposed [13]. Indeed, the embryo can efficiently implant in any tissue, independent of the stage of the cycle, with great invasion ability, while in the endometrium, it can only implant during a short period of time called the window of implantation (WOI) or window of endometrial receptivity [14,15]. In a 28-day normal cycle, the WOI occurs around 6–10 days after the LH surge and lasts about 3–6 days [14,16,17]. In cases of artificial cycles, the WOI occurs 4–7 days after the administration of progesterone [2]. The WOI is finely regulated by a plethora of factors, which include hormones, such as estrogen and progesterone, cytokines, and growth and immunomodulatory factors, all driving a series of morphological and molecular changes fundamental for a correct blastocyst-endometrial dialogue. Although the boundaries of the WOI in a 28-day cycle have been identified, the accurate assessment of endometrial receptivity in women with irregular cycles undergoing IVF is needed in order to timely transfer the embryo and reduce the risk of RIF [18]. The routine procedure to identify the day of embryo transfer in IVF is mainly based on the measurement of endometrial thickness by ultrasound, which can be ineffective as a method of predicting the risk of RIF [19,20]. Histological dating according to the Noyes criteria has been previously exploited to evaluate the morphological changes in stromal and glandular compartments along the proliferative and secretory phase of the menstrual cycle, and to identify the WOI [21]. These parameters are no longer considered predictive of endometrial receptivity, mainly due to their operator dependency [22,23]. More recently, omics approaches have been proposed as tools to identify the WOI [24,25,26,27,28]. Among these, the Endometrial Receptivity Assay (ERA) has been developed to assess the gene expression signature characterizing the receptive endometrium. However, the limits of the ERA include the assumption of signature reproducibility among cycles, the high costs, and the relatively small number of patients used to validate the assay [29,30,31].

The purpose of this review is to discuss the currently available tools used to determine embryo and endometrial parameters that dictate successful implantation and discuss the potential need for further studies, in light of a personalized approach to significantly reduce the risk of RIF.

## 2. Morphological Criteria to Identify the Best Embryo

### 2.1. Static and Morphological Embryonic Features–Cleavage Stage Embryo

Assessment of a cleavage stage embryo by using morphological characteristics considers several parameters, including cell number, the degree of fragmentation, the presence of multinucleation, and blastomere size and symmetry. Numerous studies have been conducted on the correlation between the morphology of embryos at the cleavage stage and their subsequent implantation outcomes [7]. According to the Istanbul consensus [32], the characteristics of ‘good’ embryos included 4 blastomeres on Day 2, and at least 8 blastomeres on Day 3, depending on the time elapsed post-insemination. Furthermore, blastomeres should be even sized. Moreover, embryos had to exhibit <10% fragmentation and show no signs of multinucleation. On the other hand, some studies reported a positive correlation between the live birth rate and an increment in cell count up to 8, while noting a reduction in live birth rates in embryos with more than 8 cells [33,34] Therefore, there is a general consensus about the reduced developmental potential of slow-cleaving embryos, but the developmental competence of fast-cleaving embryos remains a controversial issue.

The presence of multinucleated blastomeres in human embryos is widely recognized as a factor associated with a diminished potential of embryo development, manifested by reduced blastocyst formation, significantly lower implantation, and decreased live birth rates [35,36,37,38,39]. Therefore, the recording of this morphological characteristic should be integrated into embryo grading schemes.

### 2.2. Static and Morphological Embryonic Features–Blastocyst

The evaluation of embryo morphology is the predominant method for assessing human blastocysts worldwide [40,41,42]. This grading system incorporates various parameters such as blastocyst expansion and hatching, the appearance of the inner cell mass (ICM), and trophectoderm (TE) cohesiveness [32]. Significantly, many studies have identified a correlation between the chromosomal status of the embryo and the blastocyst morphology, with higher-quality ICM and TE being linked to increased rates of euploidy [43,44,45,46,47,48,49,50,51]. Conversely, poor quality ICM and TE are associated with elevated rates of complex aneuploidy, affecting multiple chromosomes [43,44]. In the context of embryo transfers involving vitrified-warmed embryos without genetic testing, factors such as blastocyst expansion and the grading of the TE and ICM have been linked to pregnancy outcomes. However, there remains a lack of consensus regarding the predictive value of each of these parameters, with studies yielding conflicting results about which parameter serves as the strongest predictor. Some studies showed that ICM grade had the best predictive effect [52,53,54], while others indicated that expansion stage and TE grade were stronger predictors [55,56,57]. A recent systematic review and meta-analysis revealed that embryos with a grade C ICM were correlated with a decreased rate of live births per euploid transfer compared to those with grade A/B ICM. Similarly, embryos with a grade C TE exhibited a lower live birth rate per euploid transfer compared to those with grade A/B TE. Additionally, poor quality blastocysts (<BB) were associated with reduced live birth rates per euploid transfer in comparison to high-quality blastocysts [58].

### 2.3. Morphokinetic Embryonic Features

The implementation of time-lapse technology (TLT) has facilitated an enhancement in both the frequency of observations and the dynamic monitoring of embryo development [59]. Various timings are recorded, mainly following ESHRE guidelines [60], such as the time of pronuclear fading (tPNf) and cleavage times at various stages (t2, t3, t4, etc.). Consequently, the durations of the initial three cell cycles (CC1, CC2, and CC3), as well as the period of blastocyst expansion, can be deduced from these observations. Numerous studies have explored whether these developmental timings are indicative of embryonic competence. Rienzi and coworkers observed that the duration until morulation and the quality of TE are substantial indicators for predicting live births following the transfer of euploid embryos [61]. Moreover, a recent meta-analysis, including 58 studies and over 40,000 embryos, examined a potential link between ploidy status and morphokinetic characteristics observed via TLT [62]. It was noted that aneuploid blastocysts exhibited extended durations for t8, t9, and the initiation of expansion (tEB), in addition to higher grades of fragmentation, persistent multinucleation at the four-cell stage, and blastocyst contractions. Nevertheless, due to the diverse nature of these results and the low quality of evidence, the authors recommended further research. Lastly, a retrospective study examined timings such as tPNf, t2, t3, t4, t8, tM, and tB in relation to 192 euploid single embryo transfers. Embryos resulting in live birth, euploid pregnancy loss, or no pregnancy have nearly identical morphokinetic parameters after monitoring with TLT [63].

## 3. Developmental Timing to Identify the Best Blastocyst

Full blastocyst expansion should be assessed at 116 ± 2 h post-insemination [32]. However, it has been observed that a significant number of blastocysts continue to develop beyond Day 5, with their development extending up to Day 7. Some systematic review and meta-analyses reported that clinical pregnancy and live birth rates were significantly higher following transfers of fresh or frozen-thawed blastocysts developed on Day 5 compared to Day 6/Day 7, demonstrating that blastocysts with a slower development can be of top morphological grade, euploid, and result in a healthy live birth [58,64,65]. A summary of the morphological criteria so far discussed is reported in Table 1.

## 4. Molecular Markers of Embryo Quality

### 4.1. Mitochondrial DNA (mtDNA)

The concentration of mitochondrial DNA (mtDNA) within embryonic cells has been postulated to play a key role in determining embryonic competence. Given that mitochondria originate from the oocyte and considering the established influence of oocyte quality on early embryonic development, it is plausible to suggest that mitochondrial function could significantly impact embryonic competence. This perspective aligns with the hypothesis that elevated mtDNA levels may indicate suboptimal energy production and compromised homeostasis within the embryo [66]. In fact, Fragouli et al. [67] observed a significant correlation between increased levels of mtDNA content above a threshold level and dramatically diminished clinical outcomes among euploid blastocysts. Concurrently, Diez-Juan et al. [68] confirmed these findings, suggesting a model where rising mtDNA copy numbers correlate with diminishing implantation potential.

However, these observations are not universally accepted, as other studies have reported contrasting results [69]. Victor and coworkers [70] were unable to find a relationship between mtDNA content and clinical outcomes in euploid embryos. Likewise, other studies did not identify any implantation benefits in embryos with reduced mtDNA content [71,72,73]. Moreover, the analysis of TE samples from 615 euploid human blastocysts showed that mtDNA content was not predictive of euploid human embryo reproductive competence [74]. Recently, an attempt was made to conduct a meta-analysis, but the heterogeneity in study designs, characteristics of experimental groups, analytical methodologies, and outcome measures hindered direct comparisons across studies and a real understanding of the impact of mtDNA levels on the reproductive competence of embryos [58]. These data do not support the use of mitochondrial DNA copy number in clinical decision making when selecting which embryo to transfer.

### 4.2. Cumulus Cells or Spent Media Molecular Analyses

Cumulus cells (CCs) are somatic cells closely associated with the oocyte, playing crucial roles in metabolic and signaling functions during folliculogenesis and oocyte maturation [75]. Given that oocyte competence is achieved through bidirectional signaling between the oocyte and the surrounding cumulus cells [76], and that these cells are typically discarded after oocyte retrieval, they represent a compelling and non-invasive focal point for in-depth investigation into the factors influencing preimplantation embryo quality.

Looking for molecular markers of oocyte competence in CCs constitutes a way to enhance the predictive value of conventional embryo selection. To enhance the predictive value currently obtained from standard embryo morphology assessments, these molecular markers should be able to identify oocytes which, after ART, have progressed to the blastocyst stage. More specifically, these markers should have the ability to discern those oocytes that have not only reached the blastocyst stage but are also capable of successfully establishing a pregnancy. Some studies have indicated a correlation between CCs function and embryo development. Seven genes related to CCs metabolism (CCND2, CXCR4, GPX3, CTNND1 DHCR7, DVL3, HSPB1, and TRIM28) were found to be altered at the cleavage stage in genome-wide gene expression studies [77]. Moreover, ANG, RGS2, and PLIN2 were indicated as potential predictors of blastocyst development [78]. Scarica and coworkers [79] investigated the association of CCs-related expression of a selected cluster of genes (PTGS2, CAMK1D, HAS2, STC1, and EFNB2) with embryo development to blastocyst. In particular, a strong association between the CAMK1D expression level and blastocyst formation was observed [80,81].

Other studies have explored gene expression in CCs and its relation to embryonic competence. EFNB2 and CAMK1D were suggested to be promising genes that could help to choose EFNB2 and CAMK1D were suggested to be promising genes that could help to select for transfer the embryo with the highest chance to give a pregnancy [82]. Assou et al. [83] associated NFIB reduction and BCL2L11 and PCK1 upregulation with CCs of embryos resulting in live births. Moreover, the upregulation of VCAN, PTGS2, GREM1 and PFKP in CCs of oocytes was observed in embryos leading to successful pregnancy [84]. Similarly, Wathlet and colleagues [82] described an association between pregnancy success and EFNB2, CAMK1D, STC1, and STC2 gene expression in CCs of embryos leading to successful pregnancy. Lastly, prediction models based on CCs gene expression showed upregulation of FGF12, GPR137B, SLC2A9, ARID1B, NR2F6, ZNF132, and FAM36A, and down-regulation of ZNF93, RHBDL2, DNAJC15, MTUS1, and NUP133 in the CCs of oocytes that resulted in a successful pregnancy after IVF [85].

It is noteworthy that these studies assessed pregnancy outcomes after multiple embryo transfers, without adequately accounting for the ploidy status of the embryos, limiting the applicability of these findings to current practices. In contrast, two studies conducted transcriptomic analysis on CCs from oocytes that developed into either implanting or non-implanting euploid blastocysts [86,87]. One study examined five cases per group, while another investigated 17 double embryo transfers of sibling blastocysts, yielding conflicting outcomes. Both studies identified several differentially expressed genes, but none reached statistical significance, so these genes cannot serve as reliable biomarkers of blastocyst competence.

The failure to identify transcriptome biomarkers in this analysis aligns with the results reported by Burnik Papler et al. [88]. This study, conducted using a microarray platform, similarly found no discernible differences in gene expression that could predict either oocyte fertilization or embryo implantation [88]. Moreover, recently, Sachs and colleagues [89] compared the transcriptome of CCs obtained from oocytes that resulted in pregnancy, did not result in pregnancy, led to live birth, or did not result in live birth. Although the RNA sequencing analysis did not uncover differentially expressed genes (DEGs) when comparing the transcriptomic profiles of the groups “no pregnancy” with “pregnancy”, they identified 139 DEGs when comparing the subset of “pregnancy only” with “live birth”. Notably, 28 of these differentially expressed genes were associated with clusters crucial for successful ART outcomes, such as CTGF, SERPINE2, PCK1, HHIP, HS3ST, and BIRC5 [89].

Emerging omics methodologies, including proteomics and metabolomics, are increasingly revealing distinct molecular signatures in viable gametes and embryos. These unique profiles offer potential biomarkers that may be harnessed for the purposes of developmental or viability assessment and selection. Of particular interest in ART is the secretome, those proteins that are produced within the embryo and secreted into the surrounding environment. Defining the embryonic secretome will also provide a deeper understanding of the distinctive series of events crucial for successful implantation, encompassing the essential prerequisites of the blastocyst. Given the intricate and diverse nature of the human embryo, it appears rational to anticipate a collaborative ‘omics’ approach in characterizing the human embryonic secretome [90].

Moreover, in recent years an expanding body of literature has emerged to explore the clinical applicability of spent embryo culture media (SCM) in the context of PGT-A [91,92,93,94]. Different studies demonstrated the ability to detect, extract, and amplify cell-free DNA (cfDNA) from SCM at both the cleavage and blastocyst stages. Belandres and colleagues [95] suggested enhancements to increase the precision and sensitivity of the assay prior to integrating PGT-A with SCM into clinical practice.

Additionally, it has been shown that microRNA (miRNAs) can be detected in IVF culture media, and that some of them are differentially expressed according to the fertilization method, chromosomal status, and pregnancy outcome, which makes them potential biomarkers for predicting euploidy as well as IVF success [96,97,98].

Three studies focused on miRNAs released in the SCM of euploid blastocysts, comparing those that implanted to those that did not [99,100,101]. Initially, a study involving 53 euploid single embryo transfers (SETs) found increased expression of miR-20a and miR-30c in the SCM of implanted blastocysts [100]. Nevertheless, a subsequent multicenter study that employed a tailored plate and protocol for the analysis of 10 miRNAs in 221 euploid SETs did not corroborate these findings. Although the latter study reported significant differences between non-implanted and implanted euploid blastocysts in terms of both miRNA detection and relative quantitation, when the data were adjusted for embryo morphology and day of biopsy, no significant association was confirmed [101]. The expression of miR-372 and miR-191 in embryo culture medium was found to be related to implantation failure [96], while miR-661 was successfully detected in embryonic blastocyst medium, with a higher expression in blastocysts that failed to implant [97]. Moreover, Borges and coworkers found that the expression of miR-142-3p was higher in successfully implanted embryos compared with embryos that failed to implant [102].

Recent studies have focused on the possibility of conducting PGT-A on SCM, aiming to set up a workflow to conduct non-invasive aneuploidy testing [103]. Two studies assessed outcomes following the SET of blastocysts classified as euploid via PGT-A of TE biopsy, but as either euploid or aneuploid in the SCM analysis [104,105]. A recent meta-analysis showed that SCM reported as aneuploid or euploid were associated with similar live birth and miscarriage rates per clinical pregnancy.

An additional study adopted a similar approach but complemented TE analysis with the outcome of DNA amplification from blastocoel fluid collected via blastocentesis [106]. Intriguingly, among 53 euploid SETs, the detection of DNA in the blastocoel correlated with a significantly lower live birth rate (31.5% versus 67.6%), although the miscarriage rate remained comparable. The authors suggested that this cost-effective analysis might act as a biomarker of embryo reproductive potential, indirectly revealing the impact of apoptosis or necrosis in embryonic cells, which release DNA into the blastocoel fluid. However, further research is required to substantiate this hypothesis.

In the context of spent media, extracellular vesicles (EVs) have been identified as interesting candidates able to modulate embryo development, as well as to be released by the embryos [107]. These small, membrane-bound entities released by cells have been identified in various bodily fluids, including the spent media from human embryos [108]. EVs have the capability to transport regulatory molecules such as miRNAs, mRNAs, lipids, metabolites, and proteins [109,110], reflecting the genetic makeup of the originating cells, such as the developing embryo. The membrane of EVs effectively shields enclosed cargo contents, rendering miRNAs derived from EVs more stable and reliable than free-floating miRNAs, owing to protection against RNase present in the medium [111,112].

Until now, only a few studies conducted on animal models investigated miRNAs isolated from conditioned media generated by group cultured blastocysts or degenerated embryos. These studies suggested a link with embryo quality and development [98,113]. Recent studies by Pavani et al. [114] have shed light on the selective enrichment of specific miRNAs in EVs secreted by bovine embryos reaching the blastocyst stage. The administration of synthetic forms of these miRNAs significantly enhanced the hatching capacity of blastocysts, showcasing the potential of EV-associated genetic material in influencing embryonic development. Moreover, in a study conducted by Giacomini and coworkers [108], it was demonstrated that EVs derived from human embryos obtained from ICSI carry a distinct molecular cargo, and they are internalized by endometrial cells. Additionally, EVs released by individually cultured preimplantation bovine embryos can alter the gene expression of oviduct epithelial cells [115] and endometrial cells [116]. Masoumeh Es-Haghi and colleagues demonstrated that three RNA transcripts in EVs secreted by human trophoblast spheroids were directly transferred to endometrial cells [117]. These data underline the crucial role of embryo-derived EVs in embryo–embryo and embryo-maternal communication and in the establishment of endometrial receptivity. Considering the implications for genetic diagnostics, the cargo within EVs secreted by embryos becomes a valuable source for potential biomarkers indicative of genetic health and abnormalities.

In the context of PGT, the genetic content encapsulated within EVs could provide a non-invasive and informative means of assessing the genetic status of embryos, potentially enhancing the accuracy and comprehensiveness of PGT results. By leveraging the molecular cargo of EVs secreted by human embryos, researchers may unveil new possibilities for advancing genetic diagnostics in the field of assisted reproductive technologies, paving the way for more precise and insightful genetic assessments during the preimplantation phase. A summary of the main molecular markers identifying the embryo quality is reported in Table 2.

## 5. Morphological Criteria to Assess Endometrial Receptivity

### 5.1. Endometrial Thickness (EndT)

The endometrium is a dynamic structure that undergoes repeated cycles of growth, differentiation, and apoptosis every month. The ovarian hormones, estrogen (E2) and progesterone (P4), are the main drivers of endometrial tissue plasticity. The endometrium can be divided into two layers, the stratum basalis and stratum functionalis. The latter responds to hormones, undergoes dynamic changes in cell morphology and function, sheds every month in the absence of a fertilized egg, and is the site of embryo implantation [118]. The stratum basalis is located underneath the stratum functionalis and is primarily responsible for endometrial regeneration after menstruation [119]. Indeed, the endometrial thickness changes throughout the menstrual cycle. During menstruation, the endometrial thickness is about 1–4 mm, reaching 12–14 mm in the proliferative phase under the influence of E2 [120], and a high mitotic index is observed in the epithelium, stroma, and vasculature. During the secretory phase, high levels of P4 drive the endometrium into a receptive state ready to receive the blastocyst [119], and the endometrial thickness reaches its maximum of about 16–18 mm. Since endometrial thickness (EndT) can be evaluated by minimally invasive transvaginal ultrasound, its use to establish the optimal timing for embryo transfer during IVF cycles has been proposed. In this respect, observational studies have investigated the potential association between EndT and the probability of conceiving, pregnancy outcome, and live birth rates in women undergoing IVF, often reporting conflicting results [19,121,122,123,124,125,126,127,128,129,130]. Almost a decade ago, a systematic review and meta-analysis of the published literature selected 22 retrospective and prospective studies in which different cut-offs of endometrial thickness (from 7 mm to 26.7 mm), stimulation protocols, and number of cycles were evaluated to identify a potential clinical significance of EndT at the time of embryo transfer in IVF cycles [128]. In women with endometrial thicknesses of ≤7 mm as measured at the time of ovulation, a trend toward a reduction in ongoing pregnancy and live birth rates was observed, although the threshold of statistical significance was not reached. Maternal age and the number of retrieved oocytes were taken into consideration as potential confounding factor. However, meta-regression analysis did not show a significant association with pregnancy outcome. The authors then concluded that EndT has a limited predictive value for the occurrence of pregnancy. In line with these results, a more recent retrospective study analyzing data from two large cohorts has demonstrated that EndT, measured at the time of embryo transfer, is a poor predictor of pregnancy success and live birth rates [129]. Maternal age and the number of collected oocytes were analyzed as predictive factors associated with EndT, and although EndT becomes lower with increasing maternal age, the threshold of statistical significance was not reached, while a statistically significant correlation exists between the number of retrieved oocytes and EndT. These results indicate that an evaluation of endometrial thickness should not be considered the sole determinant of the best time for an embryo transfer [129]. Contrary to the studies reported above, other studies have reported a prognostic value for EndT as a predictor of the pregnancy rate [130,131]. A retrospective study that analyzed the impact of EndT in both fresh and frozen-thaw IVF embryo transfers (ETs) demonstrated that decreased endometrial thickness had a negative effect on IVF outcomes [130]. EndT was measured on the day of ovulation trigger for fresh ETs, and at the start of progesterone treatment for frozen-thaw ETs. In fresh ETs, clinical pregnancy and live birth rates decreased significantly when endometrial thickness decreased below 8 mm, which was paralleled by an increase of pregnancy loss rate. Similar results were obtained for frozen-thaw ETs, for which an association with decreased clinical pregnancy and live birth rates was observed with endometrial thicknesses ≤ 7 mm. Interestingly, the authors found a correlation between age and the probability of EndT values ≥ 8 mm, with women aged > 40 years having the lowest probability (83.9%) compared to women age < 35 or ≥35–39 years (89.7% and 87.8%, respectively; *p* < 0.0001). However, the limitations of this study reside in the lack of information on cycle characteristics, underestimating the possibility that factors other than EndT may be responsible for the poorer pregnancy prognosis. Moreover, the analysis only included the cycles preceding embryo transfer, which might have been selected considering prognostic factors other than EndT.

To further support the hypothesis that EndT may be a poor indicator of pregnancy success, a case report described the example of a 35-year-old woman with ovarian failure, hypoplastic uterus, and atrophic endometrium after cancer treatment. She achieved pregnancy after IVF with oocyte donation [132]. Following hormonal stimulation, her endometrium reached a maximal thickness of about 3 mm, however histological analysis depicted a minimal secretory phenotype, and gene expression analysis reflected endometrial receptivity, allowing pregnancy success and a live birth. In conclusion, the thickness of the endometrium appears to have a limited ability to identify women with a low probability of conceiving after in vitro fertilization. Using the measurement of endometrial thickness to decide whether to cancel transfers and freeze all embryos or to abstain from further IVF treatments does not appear to be justified based on the available data.

### 5.2. Noyes Criteria

Morphological criteria for endometrial dating were proposed by Noyes more than 70 years ago. The Noyes criteria have been used for more than 50 years as a method to identify endometrial receptivity, and they are based on the analysis of haematoxylin and eosin-stained histological sections to identify specific morphological changes during the uterine cycle. Gland mitosis and tortuosity, the relocation of secretory vesicles in the cells of the glandular epithelium from a basal to an apical position, the presence of secreted material in the glandular lumen, stromal edema, the pseudo-decidual reaction, and leukocyte infiltration [21] are among the parameters evaluated. However, Noyes’ criteria have been widely questioned in their ability to discriminate between endometrial receptivity and a non-receptive state [133,134,135]. One of the major critiques raised concerns inter- and intra-observer variability, which may bias endometrial dating, and a comparison of endometrial receptivity assessed by Noyes’ criteria and by the expression of receptivity genes showed a poor concordance [18,22,23,134]. Nevertheless, a recent study conducted on a cohort of patients affected by recurrent implantation failure reported that the Noyes’ criteria were effective in identifying potential WOI displacements and hence providing tools for guiding a personalized frozen embryo transfer (pFET) [136]. To account for inter-observer variability, the authors compared results provided by two pathologists after a blinded evaluation of the histological specimens, reporting no significant differences. Moreover, the recent combination of histological dating with immunohistochemical localization of estrogen and progesterone receptors and the proliferation marker Ki-67 has been proposed as a better tool to identify endometrial receptivity [135]. We can therefore conclude that histological analysis implemented by the expression of specific and “unique” markers of the receptive endometrium may be used for endometrial dating to guide embryo transfer. Moreover, we can speculate that the use of artificial intelligence may correct for inter- and intra-observer variability, providing an impartial analysis of the results, thus contributing to the identification of the WOI.

### 5.3. Pinopodes

Among the morphological changes characterizing endometrial cycling, the formation of small protrusions on the apical surface of epithelial cells has been described and associated with receptivity [137,138]. The first description of pinopodes-like structures in the human endometrium was written by Johannisson and Nilsson in 1972 in an electron microscopy study [139]. The authors observed the presence of dome-like structures formed by the microvilli on the apical surface of epithelial cells of the early secretory endometrium. A role for pinopodes as markers of endometrial receptivity has been proposed by several authors, as their appearance on epithelial cells depends on progesterone levels [140] and their formation occurs on average on days 20–22 of a natural menstrual cycle, coinciding with the WOI [137,138,141]. The predictive value of pinopodes for endometrial receptivity is reinforced by results from recent clinical trials, which have correlated the development of pinopodes and their density during the WOI with the pregnancy rate, demonstrating that patients with a high pinopodes score had a higher pregnancy rate [142,143,144,145]. Nevertheless, the role of pinopodes as markers of the WOI has been questioned by some groups, mainly due to their presence not being restricted to the WOI [141,146,147]. However, beyond considering the presence and quantity of pinopodes and their coverage, their morphology and the presence of specific pinopodes-associated proteins should be also considered, in light of observed phase-dependent micro- and macroscopic changes. Indeed, an increased density of pinopodes with reduced diameters was observed in endometrial samples collected during the implantation window from women experiencing recurrent implantation failure. This phenotype was associated with the reduced expression of Ezrin and Thrombomodulin and consequent cytoskeletal alterations [145]. All together, these data suggest that a careful evaluation of pinopode density and morphology may serve as guidance to identify the WOI. However, as recently reported [148], a standardization for pinopode assessment should be highly encouraged. A schematic description of the morphological criteria used for assessing endometrial receptivity is reported in Table 1.

**Table 1:** Summary of the morphological criteria available to identify the best embryo and for endometrial receptivity assessment.

**Embryo**	**Criteria**	**Description**	**Evidence in Support**	**Evidence Against**
	Good embryo according to Istanbul consensus	At least 8 blastomeres even sized on Day 3, <10% fragmentation and no signs of multinucleation	[7,33]	[34]
	Multinucleated blastomeres	A multinucleation in Day 2 and Day 3 cleavage embryos		[35,36,37,38,39]
	ICM grading	The grading scale for ICM quality of the blastocyst	[52,53,54]	
	Expansion stage and TE grading	The grading scale for expansion and TE quality of the blastocyst	[55,56,57]	
	Developmental timing	Full blastocyst expansion should be assessed at 116 ± 2 h post-insemination	[58,64,65]	
**Endometrium**	Endometrial Thickness (EndT)	Optimal thickness for receptive endometrium of about 16–18 mm (evaluated by transvaginal ultrasound)	[124,126,130,131,149]	[122,123,128,129,132]
	Noyes Criteria	Histological criteria identifying gland mitosis and tortuosity, apical position of secretory vesicles in cells of the glandular epithelium, secreted material in the glandular lumen, stromal edema, pseudo-decidual reaction, and leukocyte infiltration	[136]	[18,22,23,133,134,135]
	Pinopodes	Evaluation of density and morphology of plasma membrane protrusions on epithelial cells projecting toward the uterine lumen on days 20–22 of a natural menstrual cycle	[137,138,140,142,143,145]	[146,147]

## 6. Biochemical Markers and Molecular Mediators of Endometrial Receptivity

In addition to morphological parameters, the expression of several molecular markers expressed during the mid-secretory phase has been studied. These include cell adhesion molecules such as integrins and cadherins, Mucin-1 (MUC-1), and LIF and LIF Receptor (LIFR) [150,151,152,153,154,155,156,157,158,159,160,161,162,163].

Among the integrins, one of the most studied is αvβ3, whose expression in the luminal and glandular epithelium is positively regulated by progesterone, epidermal growth factor (EGF), and heparin-binding EGF (HB-EGF), LIF, and negatively regulated by estradiol [164,165,166]. It has been proposed that integrin αvβ3, expressed by the luminal epithelium, may mediate the first interaction between the endometrium and the embryo trophoblast cells, suggesting a role as a potential receptor for embryo adhesion [158,165]. Studies performed by Revel et al. [167] demonstrated that patients with physiological levels of integrin β3 mRNA at Day 21 of the uterine cycle had a pregnancy rate twice that of women with lower levels, thus indicating a potential use of αvβ3 integrin as a prognostic factor for successful IVF. Moreover, the altered expression of integrin αvβ3 has been found in unexplained infertility [168,169,170,171], endometriosis [172], and polycystic ovary syndrome (PCOS) [158]. However, several cohort studies failed to observe significant differences for integrin expression in women undergoing IVF following the diagnosis of RIF, endometriosis, unexplained infertility, or tubal disease [173,174,175,176,177,178]. Additional studies are warranted to clarify this aspect, possibly comparing time at biopsy collection, type of analyses performed (e.g., protein or mRNA analyses), and sample size.

A widely proposed marker for endometrial receptivity is the high molecular weight transmembrane glycoprotein MUC1. MUC1 is a highly glycosylated protein whose expression in the luminal and glandular epithelium is regulated by progesterone and pro-inflammatory cytokines (i.e., TNF-α) [179,180,181,182,183]. While in other mammals MUC1 expression is down-regulated during the peri-implantation period in order to facilitate embryo attachment to the endometrium [184,185,186], in humans, its mRNA and protein levels increase during the secretory phase [151,152]. However, it has been reported that at the time of implantation, TNF-α released by both the blastocyst and the endometrium locally activates proteases which remove the extracellular domain of MUC1, thus favoring embryo adhesion [180,181,182]. This observation suggests that MUC1 expression is time-regulated, and its glycocalyx may guide the embryo to the correct implantation site during the apposition phase. Its removal may then allow embryo adhesion to the endometrial surface. Several studies demonstrated the altered expression of MUC1 in women undergoing RIF, recurrent pregnancy loss (RPL) [144,152,153,187], PCOS, and endometriosis [188]. Immunohistochemistry on endometrial biopsies collected on Day LH + 7 demonstrated higher levels of MUC1 in both the luminal and glandular epithelium in fertile women compared to women experiencing RIF and RPL [187]. Moreover, through scanning electron microscopy and immunofluorescence, Wu et al. have found that MUC1 is mainly localized on the surface of ciliated epithelial cells and that such expression is significantly reduced in women with reproductive failure [144]. Analysis of endometrial flushing collected at Day LH + 7, LH + 10, and LH + 13 have demonstrated high levels of MUC1 in fertile women and a significantly lower concentration in RPL patients [152,153]. A study published by Margarit et al. identified an altered expression of MUC1 in RNA and protein levels in patients suffering PCOS and endometriosis [188]. Indeed, patients with ovulatory PCOS showed levels of MUC1 higher than fertile and anovulatory PCOS patients, whereas endometriosis women showed a significant reduced expression of MUC1 compared to fertile samples. Moreover, epithelial cells isolated from the patients were treated in vitro with progesterone. Both ovulatory and anovulatory PCOS and fertile samples showed increase levels of MUC1 after treatment with progesterone, while cells from endometriosis samples did not respond to hormone stimulation [188]. In conclusion, it has been shown that the altered expression of MUC1 is associated with infertility problems, suggesting that in cases of reduced expression, the endometrium might be unable to direct the blastocyst to the implantation site or, in cases of increased expression, MUC1 carbohydrate chains might prevent the blastocyst from adhering to the endometrial surface. The most relevant biomarkers proposed to identify endometrial receptivity are listed in Table 2.

Over the last decade, growing evidence has suggested endometrial miRNAs as potential biomarkers for endometrial receptivity [189,190,191,192,193,194,195,196,197,198,199,200,201,202,203,204,205,206]. Indeed, the expression of specific miRNAs has been reported in both the epithelial and stromal compartments of the endometrium and associated with the preparation of the endometrium for implantation. In this respect, initial studies reported that up- or down-regulation of specific miRNAs in endometrial biopsies was associated to the expression of factors involved in endometrial receptivity (e.g., LIF) [189]. Subsequent studies elucidated the spatial expression of these miRNAs, showing their expression in either the epithelial or the stromal compartments and identifying the miRNA signature along the menstrual cycle in fertile women [190].

**Table 2:** Summary of the molecular markers identifying embryo quality and the biochemical markers and molecular mediators associated to endometrial receptivity.

**Embryo**	**Markers**	**Local Expression**	**Samples of Study**	**Evidence in Support**	**Evidence Against**
	mitochondrial DNA	Embryo		[67,68]	[70,71,72,73]
	CCND2, CXCR4, GPX3, CTNND1 DHCR7, DVL3, HSPB1, and TRIM28	Cumulus cells		[77]	
	ANG, RGS2, and PLIN2	Cumulus cells		[78]	
	PTGS2, CAMK1D, HAS2, STC1, and EFNB2	Cumulus cells		[79,80,81,82]	
	CTGF, SERPINE2, PCK1, HHIP, HS3ST, and BIRC5	Cumulus cells		[89]	
	miR-20a and miR-30c	Spent embryo culture media		[100]	[101]
	miR-372 and miR-191	Spent embryo culture media		[96]	
	miR-661	Spent embryo culture media		[97]	
	miR-142-3p	Spent embryo culture media		[102]	
**Endometrium**	αvβ3	Luminal epithelium	Biopsies from women with unexplained infertility, endometriosis, PCOS	[158,167,168,169,170,171,172]	[173,174,175,176,177,178]
	MUC-1	Luminal and glandular epithelium	Biopsies from RIF, RPL, PCOS, endometriosis	[144,152,153,187,188]	
	LIF/LIFR	Luminal epithelium and blastocyst	Biopsies from women with unexplained infertility	[150,156,161,162]	
	let-7 family, miR-30d, miR-183-5p, miR-192, miR-23a-3p, miR-30a-3p, miR-145, and miR-200c	Epithelial cells		[191,192,193,195,198]	
	miR21, miR-96, miR-181a, miR-200 miR-148a, miR-181b, miR-194, and miR-542	Stromal cells		[199,200,201,202,203,204,205,206]	
	miR-6821-5p, miR-483-5p 008, miR-4521, and miR-4421	Uterine fluid in secretory phase	Biopsies from RIF	[207]	
	miR-96-5p, miR-186-5p, miR-628-3p, and miR-183-5p	Uterine fluid insecretory phase	Biopsies from healthy women	[207]	

Recently, von Grothusen et al. demonstrated that different sets of miRNAs in the secretory phase uterine fluid are expressed in healthy fertile women compared to women affected by RIF [207]. Specifically, 61 differentially expressed miRNAs (34 up-regulated and 27 down-regulated) were identified in RIF samples, and their target genes were expressed by trophectoderm and endometrial epithelial cells, suggesting a potential predictive role of the miRNA signature in the regulation of endometrial receptivity and blastocyst-endometrium crosstalk. A summary of the main differentially expressed miRNAs identified in the endometrial tissue is reported in Table 2.

## 7. Transcriptomic Signature and Secretome Analysis

Given the enormous variability of the results obtained by the morphological and biochemical analysis reported above, tests based on transcriptomic analysis to identify endometrial receptivity have been developed in recent years. The importance of these tests arises from the need to identify the correct implantation window for patients undergoing IVF, in order to proceed with a personalized embryo transfer (pET). One of the first and possibly most complete of these tests is the ERA, which includes the analysis of 238 genes to classify the endometrium as non-receptive, pre-receptive, receptive, or post-receptive [24]. The driving hypothesis underlying the ERA is the consideration that gene expression analysis may have greater objective accuracy than morphological and/or biochemical analyses to identify the WOI [25]. In this respect, several studies have been carried out to validate the use of the ERA to increase the probability of clinical pregnancy and the live birth rate after ET and to study the differential gene expression between healthy controls and women undergoing RIF [26,27,28,29,30,31,208,209,210]. However, limitations exist. Indeed, most of these studies are retrospective, and they generally include small numbers of patients and due to the surgical procedure necessary to collect the endometrial sample, they report on embryo transfers that occurred in a subsequent cycle. This latter aspect raises critiques, since it relies on the assumption that the uterine molecular signature of one cycle is reproduced with no variations in a subsequent cycle. So far, no proof exists to support such an assumption, and hence analyses performed in one cycle might not guarantee the correct identification of the WOI in the next cycle. Alternatives to the ERA test have been suggested [211,212,213,214]. Indeed, high throughput RT-qPCR has been applied to compare the expression of 184 genes involved in endometrial receptivity and immunity in endometrial biopsies from fertile and sub-fertile patients. Using principal component and discriminant function analyses, among the 85 differentially expressed genes, 40 genes were identified to classify the endometria as receptive or non-receptive and to program a personalized embryo transfer [211,212]. Using a similar approach, He et al. developed an RNA-Seq-based endometrial receptivity test (rsERT) to identify the WOI in RIF patients, and reported a significant increase in implanted embryos, intrauterine pregnancy, and live birth rate in patients undergoing rsERT-guided pET, compared to patients undergoing conventional ET [213]. More recently, a new test based on Targeted Allele Counting by sequencing (TAC-seq) and named beREADY has been proposed for endometrial dating [214]. The analysis of 68 endometrial receptivity genes in endometrial biopsies of healthy volunteers allowed the researchers to identify pre-receptive (proliferative and early secretory samples), receptive (mid-secretory samples), and post-receptive (late secretory samples) stages of the endometrium, thus contributing to the identification of WOI displacement in RIF patients and when to proceed to a personalized embryo transfer.

All of the above-mentioned tests have been designed to identify the endometrial fingerprint associated with the WOI, collecting tissues in the secretory phase. However, this approach inevitably requires ET to be performed in a subsequent cycle, hence facing the potential cycle to cycle variability bias. To overcome this, some studies have investigated the possibility of identifying markers predictive of implantation competence by studying gene expression in the proliferative phase and performing embryo transfer in the same cycle [215,216]. In this respect, Zhou et al. found 218 genes differentially expressed between women who achieved a clinical pregnancy and those who did not, thus suggesting that the transcriptomic analysis of endometrial samples in the proliferative phase after a stimulated ovarian cycle may provide crucial information prior to fresh ET [215]. More recently, a combination of both transcriptome analysis of endometrial samples in the proliferative phase and secretome analysis of endometrial stromal cells (hESC) isolated from biopsies have been performed [216]. Differently from what reported by previous studies [215], transcriptomic analysis did not reveal significant changes in gene expression with respect to the outcome of embryo transfer; however, secretome analysis of 45 cytokines in media from in vitro decidualized hESC obtained from these biopsies revealed interesting results. Indeed, hESCs were previously shown as key regulators of implantation and sensors of embryo quality [217,218,219], and in vitro decidualized hESC showed a different secretome signature when obtained from the control as opposed to the implantation failure groups [216]. This study highlighted the potential of hESCs isolation in proliferative phase and the analysis of their secretome as a clinical tool in ART, in order to perform ET in the same uterine cycle of the biopsy.

The importance of the endometrial secretome has been also recently studied by Gurung et al. [220]. Using western blot and liquid chromatography-tandem mass spectrometry, the authors demonstrated that the exosome fraction, was better able to stimulate adhesion and outgrowth of trophoblast spheroids or favor mouse blastocyst development and hatching than total or soluble secretome from the ECC1 cell line. Although in this study primary human cells were not used, the reported results suggest the importance of the endometrial epithelial secretome in supporting implantation and embryo growth and the potential use of exosomes to improve implantation success [220].

Secretome analysis has been also performed on uterine fluid, allowing the identification of proteins and secreted factors differentially expressed between the proliferative and secretory phases of the uterine cycle [221,222,223,224]. The isolation of extracellular vesicles from the uterine fluid demonstrated the presence of proteins and nucleic acids capable of increasing sperm mobility and acrosome reaction, oocyte maturation, and embryo endometrial crosstalk [225,226,227]. More recently, small extracellular vesicles or exosomes were isolated from uterine lavage to study their content in the different phases of the uterine cycle of fertile patients [224]. The analysis demonstrated a relevant presence of proteins involved in immune response, antioxidant activity, and lipid metabolism in the proliferative phase, while in the secretory phase mitochondrial activity proteins were predominant. Antioxidant activity is important to protect the embryo, and an increase in ROS has been associated with implantation failure or pregnancy loss [228,229]. Indeed, the analysis of exosomes from infertile patients showed a down-regulation of proteins involved in antioxidant function, supporting the hypothesis that these small extracellular vesicles protect the embryo from the changes occurring in the uterine microenvironment during the menstrual cycle [224]. Moreover, in vitro experiments, using trophoblast spheroids to mimic the embryo, confirmed that exosomes released during the secretory phase have a greater content of factors able to increase trophoblast invasion ability and that these proteins are less expressed in infertile patients, suggesting their involvement in embryo implantation and the establishment of a successful pregnancy [224]. A summary of the main data described in this paragraph is reported in Table 3. 

**Figure 1:**
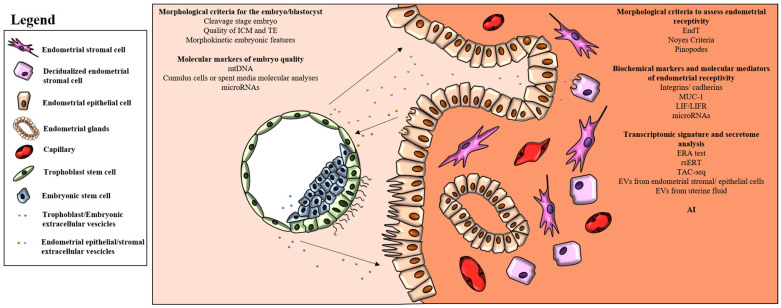
Schematic representation of the morphological and molecular criteria used to improve the chances of successful embryo implantation. AI should be applied to integrate all data related to the embryo and endometrium in order to increase the efficiency of ART.

A schematic representation of all the data reported in the above sections is reported in Figure 1.

## 8. Artificial Intelligence in ART

An expanding field of machine learning that has been increasingly used in recent years is artificial intelligence (AI). Thanks to specific algorithms and statistical analyses, AI is able to match different data and variables present in a database. AI is a powerful technological tool applicable in several fields, including ART. Indeed, AI can be applied to the evaluation of gametes to be used for in vitro fertilization. In this respect, specific algorithms have been developed for oocyte selection by integrating time-lapse data and gene expression or transcriptomic analysis in order to minimize the number of oocytes to be fertilized, avoiding the production of a surplus of cryopreserved embryos [230,231,232,233,234]. AI has also been exploited for sperm selection, integrating data on motility, concentration, viability, and morphology [231,235]. In addition, AI has been recently used for assessing embryo viability, quality, and developmental stage (from cleavage stage embryo to blastocyst), in order to select the best embryo to transfer, vitrify, or biopsy [231,236,237,238]. Considering the significant correlation between blastocyst quality with euploidy and implantation, AI-powered time-lapse microscopy appears to be a promising non-invasive and non-static strategy for the morphological assessment of blastocysts. Indeed, AI would make it possible to eliminate high operator subjectivity, allowing a better evaluation of a dynamic and complex process that cannot be appreciated through static assessment [239,240,241]. A further application of AI concerns its potential use in the design of therapeutic protocols. For example, AI has been proposed as a tool to identify the starting and total doses of FSH to be administered in order to maximize oocyte maturation, allowing the clinician to adopt a patient-personalized therapeutic approach.

**Figure 2:**
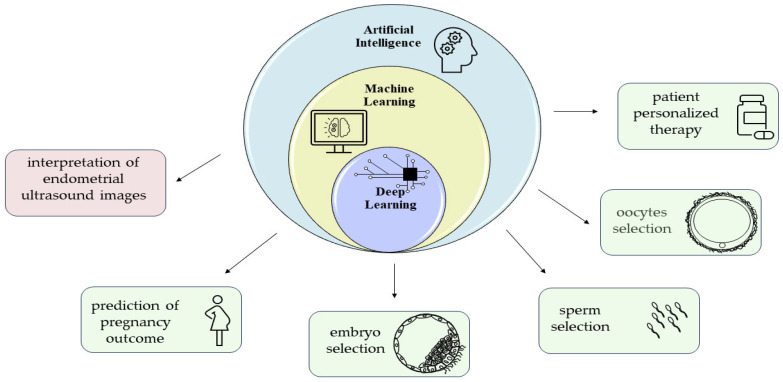
Schematic summary of possible applications of AI in ART.

Although increasingly used in ART clinics, the application of AI to the study of endometrial receptivity remains scant. As a matter of fact, there are still very few articles in the literature that used AI for endometrial staging, possibly due to the need to combine the many different aspects defining a receptive versus a non-receptive endometrium. Nevertheless, given the wide possibilities that AI offers, it can be hypothesized that the future will see a rapid development of algorithms for this purpose. Indeed, deep learning approaches have recently been applied to the interpretation of endometrial ultrasound images [242,243]. However, machine learning is still in an early stage, and further studies are needed to generate a greater quantity and quality of data to improve deep learning’s performance in clinical activities [237]. A summary of what has been so far discussed is reported in Figure 2.

## 9. Conclusions

Human reproduction remains a fairly inefficient process, and the continuously increasing maternal age at the time of first pregnancy further increases the risk of reproductive failure. Assisted reproductive technology represents a valid aid to increase pregnancy success. However, specific conditions (e.g., recurrent implantation failure) still represent a limit for ART. Over the last decade, significant improvements in morphological and molecular tests have helped to partially overcome these limitations. The implementation of machine learning techniques could be the key, allowing the integration of data relative to the endometrium and its receptivity, and the refinement of the criteria to select competent embryos.

## Figures and Tables

**Table 3:** Summary of the available assays to identify endometrial receptivity.

Assay	Outcome	Samples of Study	References
Endometrial ReceptivityAssay (ERA)	Identification of 238 genes to classify the endometrium as non-receptive, pre-receptive, receptive, or post-receptive	Biopsies from healthy fertile women, RIF, hydrosalpinx, and sub-fertile	[25,26,27,29,30,31,33,209,210]
High throughput RT-qPCR	Identification of 40 genes to classify the endometrium as receptive or non-receptive	Biopsies from fertile and sub-fertile patients	[211,212]
RNA-Seq-based endometrial receptivity test (rsERT)	Identification of 175 predictive genes to identify the receptive endometrium	Biopsies from RIF	[213]
Targeted Allele Countingby sequencing (TAC-seq)	Identification of 68 endometrial receptivity genes to identify pre-receptive, receptive, and post-receptive endometrium	Biopsies from healthy volunteers and RIF	[214]
Transcriptome analysis (gene chip analysis)	Identification of 218 genes in proliferative phase to perform fresh embryo transfer in the same cycle	Biopsies from women undergoing fresh IVF-ET cycles	[215]
Secretome analysis	Identification of 45 cytokines in media from in vitro culture of decidualized hESC	Biopsies from fertile patients and RIF	[216]
Secretome analysis	Identification of the exosome fraction able to stimulate adhesion and outgrowth of trophoblast spheroids	ECC1 epithelial cell line	[220]
Secretome analysis	Identification of proteins, secreted factors, nucleic acid, and sEV and exosomes differentially expressed between proliferative and secretory phases of endometrial sample	Uterine fluid and/or trophoblast cell line	[221,222,223,224,225,226,227]

## Abbreviations

ART	assisted reproduction technology
IVF	in vitro fertilization
PGT	preimplantation genetic testing
RIF	recurrent implantation failure
WOI	window of implantation
LH	luteinizing hormone
ERA	endometrial receptivity assay
ICM	inner cell mass
TE	trophectoderm
TLT	time-lapse technology
tPNf	time of pronuclear fading
tEB	time for expansion blastocyst
tM	time to morula
tB	time to blastocyst
mtDNA	mitochondrial DNA
CCs	cumulus cells
CCND2	cyclin D2
CXCR4	CXC chemokine receptor 4
GPX3	glutathione peroxidase 3
CTNND1	catenin delta-1
DHCR7	7-dehydrocholesterol reductase
DVL3	disheveled dsh homologue 3
HSPB1	heatshock 27 kDa protein 1
TRIM28	tripartite motif-containing 28
ANG	angiogenin
RGS2	regulator of G-protein signaling 2
PLIN2	perilipin 2
PTGS2	Prostaglandin-endoperoxide synthase 2
CAMK1D	calcium/calmodulin-dependent protein kinase 1D
HAS2	hyaluronic acid synthase 2
STC1	stanniocalcin-1
EFNB2	ephrinB2
NFIB	nuclear factor 1 b
BCL2L11	BCL2 like 11
PCK1	phosphoenolpyruvate carboxykinase 1
VCAN	versican
GREM1	gremlin 1
PFKP	phosphofructokinase, platelet
STC2	stanniocalcin-2
FGF12	fibroblast growth factor 12
GPR137B	G-protein–coupled receptor 13b
SLC2A9	Solute carrier family 2 (facilitated glucose transporter), member 9
ARID1B	AT-rich interactive domain 1B (SWI1-like)
NR2F6	Nuclear receptor subfamily 2, group F, member 6
ZNF132	Zinc finger protein 132
FAM36A	Family with sequence similarity 36, member A
ZNF93	Zinc finger protein 93
RHBDL2	Rhomboid, veinlike 2 (Drosophila)
DNAJC15	DnaJ (Hsp40) homologue, subfamily C, member 15
MTUS1	Microtubule-associated tumor suppressor 1
NUP133	Nucleoporin 133kDa
DEGs	uncover differentially expressed genes
CTGF	Connective tissue growth factor
HHIP	Hedgehog interacting protein
HS3ST	Heparan sulfate glucosamine 3-O-sulfotransferase 1
BIRC5	Baculoviral IAP Repeat-Containing 5
SCM	spent embryo culture media
cfDNA	cell-free DNA
miRNA	microRNA
SET	single embryo transfers
EV	extracellular vesicles
ICSI	intracytoplasmic sperm injection
EndT	Endometrial Thickness
E2	estrogen
P4	progesterone
pFET	personalized frozen embryo transfer
MUC-1	Mucin-1
LIF1	leukemia inhibitory factor
LIFR	LIF Receptor
EGF	epidermal growth factor
HB-EGF	heparin-binding EGF
PCOS	polycystic ovary syndrome
TNF-α	tumor necrosis factor α
RPL	recurrent pregnancy loss
rsERT	RNA-Seq-based endometrial receptivity test
TAC-seq	Targeted Allele Counting by sequencing
hESC	endometrial stromal cells
ROS	reactive oxygen species
AI	artificial intelligence
FSH	follicle-stimulating hormone
